# Supplementary material for: The Dialogue study: Protocol for a randomized clinical trial evaluating the efficacy of virtual reality-based psychotherapy plus treatment as usual versus treatment as usual for eating disorders
Source: PLoS One. 2025 Jul 28;20(7):e0319875. doi: 10.1371/journal.pone.0319875 (PMC12303276; doi:10.1371/journal.pone.0319875)
Supplement: S1 File — The latest version of the protocol approved by the Committee on Health Research Ethics of the Capital Region of Denmark (reference number: H-22067692). (DOCX) [file pone.0319875.s001.docx]

The Dialogue Study

*En undersøgelse af effekten af virtual reality-baseret behandling til patienter med en spiseforstyrrelse: et randomiseret, klinisk forsøg.*

Indhold

[Aim: 4](#_Toc120534639)

[Background: 4](#_Toc120534640)

[Hypotheses: 5](#_Toc120534641)

[Method: 5](#_Toc120534642)

[Procedure, participants, and eligibility: 5](#_Toc120534643)

[Treatments: 6](#_Toc120534644)

[Adverse events: 6](#_Toc120534645)

[Instruments and outcome measures: 6](#_Toc120534646)

[Statistical analyses and power calculation: 7](#_Toc120534647)

[Randomization and blinding: 8](#_Toc120534648)

[Ethics: 8](#_Toc120534649)

[Organisation 8](#_Toc120534650)

[Feasibility: 8](#_Toc120534651)

[Clinical relevance: 9](#_Toc120534652)

[Dissemination 9](#_Toc120534653)

[Time and work Schedule: 9](#_Toc120534654)

[Dansk tillæg til forsøgsprotokol 10](#_Toc120534655)

[Informeret samtykke og rekruttering 10](#_Toc120534656)

[Kontakthyppighed 11](#_Toc120534657)

[Kriterier for diskontinuation 11](#_Toc120534658)

[Procedure for discontinuation 11](#_Toc120534659)

[Undersøgelser i studiet 11](#_Toc120534660)

[Symptom- og funktionsniveau 11](#_Toc120534661)

[Behandlingsmodaliteter 11](#_Toc120534662)

[Medicinering 11](#_Toc120534663)

[Forsøgets bivirkninger, risici og ulemper 12](#_Toc120534664)

[Databehandling 12](#_Toc120534665)

[Journaloplysninger 12](#_Toc120534666)

[Erstatningsordning 12](#_Toc120534667)

[Videnskabsetisk redegørelse 12](#_Toc120534668)

[Fordelene for patienterne 13](#_Toc120534669)

[Ulemperne for patienter 13](#_Toc120534670)

[Studiets potentiale 13](#_Toc120534671)

[Økonomiske forhold og klausuler 13](#_Toc120534672)

[Initiativtager 13](#_Toc120534673)

[Vederlag 14](#_Toc120534674)

[Offentliggørelse 14](#_Toc120534675)

[Organisation 14](#_Toc120534676)

[Gennemførlighed 14](#_Toc120534677)

[References 15](#_Toc120534678)

# Aim:

The Dialogue study is the first trial to evaluate the effect of an innovative short-term, virtual reality (VR)-based therapy for patients with an eating disorder (ED), targeting the power and control of their ED. The objective is to identify whether this VR-based therapy can reduce symptoms, improve quality of life, and be cost-effective in patients with an ED. Additionally, the study will exploratorily investigate whether the intervention can improve the blood marker profile, and finally, whether indications on the neuroplastic potential of the intervention can be established.

# Background:

Eating disorders (ED) can be defined as pathological eating habits and a tendency to overestimate weight and body shape^1^. It affects at least 9% of the global population^2^ and has profound physical and psychological costs for the affected individual^3^ comprising a high relapse rate and highest mortality rate of any mental illness^4,5^. ED are associated with a significant economic burden in terms of health care costs and lower employment rates^6^. ED interventions are recommended to comprise psychological therapies^7^ but these are, however, often of a long duration (up to 18 months)^8^. Consequently, there is considerable interest in the development of novel therapies for ED which are shorter and more effective. The majority (94%) of patients with an ED report hearing an internal voice commenting in second or third person on shape, weight, eating, and self-worth^9^. The ED voice typically emerges at illness onset were it is often perceived as supportive, but over time it becomes critical and dominant, and increasingly expects destructive eating behaviors^10^. The experience of a more powerful ED voice is related to more serious compensatory behavior and longer illness duration^10^. Recently it has become of scientific interest to develop interventions aiming at diminishing the ED voice^9,10^.

Within psychotic disorders, a targeted VR-based therapy, termed avatar therapy, has proven highly effective (effect size 0.8) in reducing the power of the psychotic voice (auditory hallucination) and consequently alleviating the associated distress^11,12^. Our research group is conducting a large-scale randomized clinical trial evaluating this VR-based therapy for auditory hallucinations^13^. Mirroring international findings, our preliminary qualitative data reveal this short-term (i.e., 7-sessions) therapy to be highly effective in decreasing voices. Building on this evidence, we have developed a modified version of the treatment protocol targeting patients with an ED. The therapy involves the participant creating a virtual embodiment (avatar) of the ED voice, enabling the person to engage in real-time “face-to-face” dialogue with his/her ED voice. The patient is encouraged to stand up to the ED voice to gain increased power and control over it. This is expected to translate into reduced symptoms and increased functioning and quality of life. Being in dialogue with the eating disorder voice in VR, as opposed to engage in role play with the therapist (the therapist being the ED voice), is expected to be more powerful as growing evidence suggests that greater immersion in a VR system increases both the feeling of presence and emotional arousal^14^. Sense of presence offers access to emotional meaning-making (‘hot’ cognition) and has been linked to greater clinical improvement^14^. Also, creating and interacting with the avatar together with a therapist acts as a powerful validation of the otherwise private experience of the persons “eating disorder voice”.

To evaluate the comprehensive impact of the VR-based treatment on eating disorders (anorexia and bulimia), it is essential to measure plasma nutritional pre/post intervention. These markers, spanning vitamins, trace elements, metabolites, hormones, and proteins, are key to objectively determining changes in nutritional status brought about by the treatment. Successful interventions typically manifest as normalization or improvements in these markers. For example, recovery from protein-energy malnutrition is indicated by stabilized or increased levels of plasma proteins like albumin and prealbumin. Additionally, enhancements in the levels of fat-soluble vitamins (A, D, E, and K) and essential trace elements such as zinc and iron signal the replenishment of nutrient stores, vital for metabolic repair and physiological function.

The observed changes in these biomarkers not only highlight the intervention's direct effect on nutrient absorption and utilization but also encapsulate broader dietary and health improvements. By scrutinizing these variations, our study seeks to uncover the extensive benefits of VR-treatments in addressing the nutritional deficits common in eating disorders. Through a detailed examination of plasma markers, we aim to assess the effectiveness of this intervention comprehensively, offering insights that could potentially transform approaches to nutritional recovery for individuals with anorexia.

Additionally, the eating disorder research field has witnessed an increasing interest in elucidating neurobiological underpinnings of eating disorders along with the potential for achieving brain-related changes as part of an intervention ^15^. In this context MRI-scans can provide valuable insight into understanding treatment mechanisms, paving the way for more effective and targeted interventions. Reviews find alterations in both functional and structural changes in eating disorders with MRI studies providing most prominent evidence on gray and white matter reductions correlating with malnourishment and relating to recovery. In fMRI studies the most pronounced findings are increased activation of the amygdala and altered activation in the singular cortex^16,17^. While no published studies have utilized MRI to investigate brain related outcomes measures of a virtual reality-based treatment, or any therapeutic interventions, in eating disorders, there is evidence from anxiety disorders on a VR-intervention resulting in a decrease in brain activity after the intervention in specific structures (e.g., prefrontal and frontal cortex). Hence, adding MRI-scans on a subsample of the study participants may provide a signal on how the intervention works.

# Hypotheses:

- VR-assisted psychotherapy as add on to regular treatment will be superior to specialized individualized treatment in reducing eating disorder symptoms, comorbid symptoms, and improve quality of life.
- VR-assisted psychotherapy as add on to regular treatment will be cost-effective in treating eating disorder symptoms.
- VR-assisted psychotherapy will improve specific and unspecific biomarkers of nutritional health.
- VR-assissted psychotherapy will show indications on improvements in structural and functional brain related measures e.g.:
  - Task based fMRI (executive control, cognitive flexibility)
  - Gray and white matter reductions

## Method:

The study is a randomized, assessor-blinded parallel-groups superiority trial.

## Procedure, participants, and eligibility:

A total of 96 patients will be allocated to either 7 sessions of virtual reality-assisted psychotherapy + standard treatment or specialized treatment. All participants will be assessed at baseline and at 3- and 6 months follow-up. The study will enroll participants from the eating disorder out-patient facility in the Mental Health Services in the Capital Region and Region Zealand. Inclusion criteria: Age 18 – years, ability to give informed consent, a diagnosis of eating disorder, recognizing having an eating disorder voice, a score of ≥ 2.77 on the EDE-Q assessing level of eating disorder symptoms. Exclusion criteria: Unable to identify an eating disorder voice, a diagnosis of organic brain disease, a command of spoken Danish or English inadequate for engaging in therapy, comorbid psychosis, psychotic depression, active suicidal ideations.

## Treatments:

**Virtual reality-based therapy (experimental group):**

Patients will be offered 7 individual sessions of VR-based therapy conducted by a therapist experienced in both ED and VR-based therapies (including avatar therapy). Initially, the participants create a virtual avatar that corresponds to their perception of their “ED voice”. Additionally, a voice transformation program transforms the voice of the therapist to match the “ED voice” as experienced by the patient. In the following sessions, the therapist initiates and supports a dialogue between the participant and the avatar (symbolizing the ED). Each therapy session lasts 60 minutes of which approximately 15 minutes is spent in dialogue with the avatar. The remaining time is used on preparing the patient for the confrontation with the avatar, evaluating the interaction, and general cognitive behavioral techniques to reduce the symptoms of ED. The participant will wear VR headset during treatment and watch and talk with the avatar shown in front of him/her.

**Specialized treatment (comparison group):**

The comparison group will be offered highly specialized treatment provided by interdisciplinary health professionals. It includes 7 sessions of individual or group psychotherapy and dietary guidance. It is typically managed by the specialized psychiatric outpatient facility in Mental Health Center Ballerup as well as by the specialized psychiatric outpatient clinic in Mental Health Center Zealand. The therapy and counselling offered is expected to be better than standard care.

## Adverse events:

Virtual reality therapy is generally well tolerated and with minimal or none side effects or adverse events^18^. There are, though, few reports on cyber sickness caused by the VR therapy^19^. Side effects and adverse events will be monitored and recorded throughout the study period, as well as complaints about therapy. The following are considered as adverse events: 1) hospital admissions; 2) suicide attempts; 3) any violent incident necessitating police involvement (whether victim or accused); 4) self-harming behaviour; 5) all deaths. Any adverse events will be reported to the Committee on Health Research Ethics of the Capital Region Denmark. It will be emphasized that the participants are free to leave the program at any time without it having any consequences for their further psychiatric treatment.

## Instruments and outcome measures:

**Primary outcome:** eating disorder symptoms measured with the Eating Disorder Inventory^20^. **Secondary outcomes:**. Beliefs about voices questionnaire, the Hospital Anxiety and Depression Scale, Identity and eating disorder questionnaire (IDEA), SOCQ-ED (stages of change questionnaire for eating disorders), Experience of an Anorexic Voice Questionnaire (EAVE-Q).

**Explorative outcomes:** eating disorder voice characteristics assessed with a modified version of the Psychotic symptoms rating scale, auditory hallucinations^21^. Body Shape Questionnaire^22^, Eating Disorder Quality of Life Scale^23^, the self-compassionate scale^24^ EQ-5D^25^ (for cost-effectiveness analyses also comprising register-based data), Client Satisfaction Questionnaire^26^, Simulator Sickness Questionnaire ^27^, , the General Self-Efficacy Scale^28^ ^29^, Embodiment Questionnaire^30^, Behaviour Rating Inventory of Executive Functioning^31,32^, Presence Questionnaire^33^. Childhood Trauma Questionnaire^34^ and Dysfunctional Attitude Scale^35^, QPR-15 (questionnaire about personal recovery). Additionally, blood collection will assess potential biomarkers of nutritional health. Finally, MRI-scans will provide a potential signal on the changes in brain activity that may be associated with the therapeutic effect.

## MRI-scans

A subsample of 30 participants (15 from the VR-intervention group and 15 from the control group) will be assessed with functional and structural MRI and baseline and at cessation of treatment (3 months follow-up). MRI-scans will be conducted at Glostrup Hospital, Center for Functional Imagining and conducted by staff trained in MRI-procedures and includes a MP2RAGE T1 structure image (volume and grey/white matter tissue), MRS (spectroscopy), task-based fMRI, resting-state fMRI. Each scan will last approximately 90 minutes including breaks.

## Blood collection and storage:

At baseline and 3-months follow-up (cessation of treatment) patients will be asked to give a blood sample. Blood (approximately 20 mL) is drawn from a cubital vein by standard needle by an authorized laboratory technician, nurse, or doctor. Blood is collected in standard blood tubes. Whole blood and plasma will be collected for further analysis. Biochemistry markers of nutrition including albumin, hemoglobin, mean corpuscular volume (MCV), ferritin, vitamin D, calcium, magnesium, phosphate, zinc, vitamin B12, folate, total lymphocyte count (TLC) will be analyzed according to best clinical practice. Furthermore, hormonal, metabolite and protein panels will be measures as an exploratory outcome to identify known and unknown markers of nutritional health these will be done using immune-based and mass-spectrometry based approaches.

The biological material will be kept in a research biobank at secured freezer at Psychiatric Centre Gentofte until analyzed. The research biobank will apply for permit by ‘Videnscenter for Datasikkerhed´ in line with current legislation. The biobank will keep the biological material until the study is completed and all analyses has been conducted. Hereafter the materials will be safely destroyed, no later than 31st of May 2031.

## Statistical analyses and power calculation:

The study will use a mixed-methods design. Comparisons between the two groups on continuous outcomes will be carried out with a generalized linear model adjusted for stratification variables, potential baseline imbalances and skewed attrition. Linear mixed model analyses with repeated measurements and an unstructured covariance matrix will assess the interaction term between time and intervention. All analyses will be according to the intention-to-treat principle. Qualitative analyses will elucidate patients experience with therapy. Our primary hypothesis is difference in treatment effect between the two groups as measured by the EDI-BS. Our primary hypothesis is difference in treatment effect between the two groups as measured by the EDI-BS. A previous study found a EDI-BS score of 21(SD=2.5) in control condition and a score of 24.5 (SD=2.6) in the experimental condition [30]. For Dialogue, we consider the minimal clinically important difference to a true difference in the experimental and control means of 1.5. Calculating effect sizes reveal 48 subjects are required in each group to detect the expected difference in a t-test with 80% power at the 0.05 significance level using a two-sided hypothesis.

## Randomization and blinding:

Randomization will be centralized and computerized with a concealed randomization. Block size will be unknown to the investigators and clinicians. Assessors will be blind to treatment allocation.

# Ethics:

The study will be approved by the Regional Health Ethics Committee, Data Protection Agency, and registered at ClinicalTrial.gov.

# Organisation

The trial will be carried out at VIRTU research group (headed by associate professor Louise Birkedal Glenthøj, LBG) that has four trials evaluating the use of VR-therapies for psychiatric disorders (affective- and psychotic disorders). VIRTU was recently nominated Innovation of the year by the Danish clinical research prize. VIRTU is part of Copenhagen Research Centre for Mental Health, head Prof. Merete Nordentoft that has been conducting clinical studies for two decades. The Dialogue trial will be conducted in close collaboration with Prof. Nadia Micali, Head of Eating Disorder Research Unit, Psychiatric Center Ballerup, Dr. Tom Ward, Kings College London, specialized in delivering avatar therapy, and Prof. Stig Poulsen, Institute for Psychology Copenhagen University, expert in treating eating disorders. The trial is supported by “Foreningen Spiseforstyrrelser og Selvskade”.Prof. Henrik Larsson Center for Functional Imaging, Glostrup Hospital will collaborate on the MRI-scans, and Nicolai J. Wewer Albrechtsen Associate Professor at NNF Center for Protein Research, University of Copenhagen Staff Consultant at Department of Clinical Biochemistry, Bispebjerg Hospital. Emma Ries, psychologist experienced in working with ED and conducting VR-based therapies, will deliver the VR-based intervention supervised by Dr. Tom Ward. Nina K Hansen (NKH), research assistant on ongoing VR-based clinical trials at VIRTU, will be PhD student responsible for data collection, academic products, and study presentations. The following papers are planned to form part of the PhD-study and submitted to international journals:

1. *The Dialogue Study: the effect of a virtual reality-assisted therapy targeting the power and control of an eating disorder voice versus specialized individualized treatment: study protocol for a RCT 2) The effect of virtual reality-assisted therapy for patients with an eating disorder: results from the Dialogue study, 3) The cost-effectiveness of a short-term, virtual reality-based therapy for eating disorders.*
2. *MRI based changes following a brief virtual reality-based intervention for eating disorders: a proof-of-concept study.*
3. *Changes in nutrition blood markers following a virtual reality based intervention for anorexia and bulimia.*
4. *Elucidating functional and structural brain changes in a study utilizing virtual reality-based intervention for eating disorders.*

# Feasibility:

There is an established collaboration with eating disorder unit at Mental Health Center Ballerup that yearly treats approx. 1200 adult patients with an ED, hence it is highly realistic to recruit the target number of 96 patients within 1.5 years. The therapy has been evaluated by service-users that have participated in finalizing the design of the intervention. Based on experience from our ongoing VR-trials (more than 300 patients), VR-interventions are considered appealing, engaging, and tolerable. The VR-software used in the Dialogue study is well-tested. The VR-equipment and software are already acquired.

# Clinical relevance:

The use of VR in mental health treatment has great potential with promising results on efficacy and cost-effectiveness^36–38^. If this short-term (7-sessions) VR-based treatment proves effective and cost-effective in treating eating disorders, there is huge potential in terms of scalability and implementation into psychiatric practice in all of Denmark where it may benefit a significant target group.

# Dissemination

Study results will be disseminated to the scientific audience at relevant conferences and the scientific papers submitted to relevant psychiatric journals (e.g. International Journal of Eating Disorders). Results will be presented to the public through LMS, Psychiatry Foundation etc.

# Time and work Schedule:

Ethics and data protection approvals: **Jan - Jun 2023**.

Service-user feedback: **Apr - Oct 2023**

Recruitment and baseline assessments: **Jul 2023 – Dec 2024**

3- and 6-months follow-up: **Oct 2023 – Jun 2025**

Analyses, writing of manuscripts: **Apr 2025 – Mar 2026**

|  | 2023 | | | | 2024 | | | | 2025 | | | | 2026 | |
| --- | --- | --- | --- | --- | --- | --- | --- | --- | --- | --- | --- | --- | --- | --- |
| Approvals: Data Protection and Ethical Committee. REDCap database | x | x |  |  |  |  |  |  |  |  |  |  | |  |
| Service-user feedback on therapy, finalizing the intervention to be tested |  | x | x |  |  |  |  |  |  |  |  |  | |  |
| Trial initiation: Recruitment and baseline assessment |  |  | x | x | x | x | x | x |  |  |  |  | |  |
| 3- and 6-months follow-up |  |  |  | x | x | x | x | x | x | x |  |  | |  |
| Analyses, writing of manuscripts |  |  |  |  |  |  |  |  |  | x | x | x | | x |

# Dansk tillæg til forsøgsprotokol

## Informeret samtykke og rekruttering

Rekruttering af patienter vil ske i samarbejde med sundhedspersonale fra specialiseret, voksenpsykiatrisk, spiseforstyrrelsesenhed på Psykiatrisk Center Ballerup, Region Hovedstaden samt Region Sjællands enhed for spiseforstyrrelsesbehandling. Personalet fra spiseforstyrrelsesenhederne formidler kontakten til de projektansvarlige, såfremt patienten indvilliger heri*.*

Alternativt kan der rekrutteres patienter via Landsforeningen Spiseforstyrrelser og Selvskade. Dette vil foregå ved, at forskningsassistent holder oplæg i foreningen og hvor interesserede kan henvende sig mhp. udlevering af projektmateriale. Derudover gøres der opmærksom på projektet på foreningens sociale medier, hvor interesserede kan henvende sig til forskningsassistent mhp. at høre yderligere om evt. projektdeltagelse. Yderligere rekrutteres fra andre kommunale, regionale og private tilbud til personer med spiseforstyrrelser (f.eks. Københavns Kommunes – Robus, private behandlingsenheder, f.eks. Kildehøj Privat Hospital, Kompetencecenter for spiseforstyrrelser og Hejmdal Privat Hospital, spiseklinikken**).**

Der kan ligeledes rekrutteres selv-henvendere fra online-portalen forskning.nu, via sociale medier (Facebook, Youtube, Instagram og LinkedIn) i form af opslag om projektet samt via ophæng af plakater og foldere på offentlige lokationer (uddannelsessteder, fitnesscentre/sportshaller, praktiserende læger og -psykologer, biblioteker og rådgivningstilbud). Som alternativ til en hjemmeside oprettes en facebookside, hvor interesserede kan finde information om projektet. Ved rekruttering via de sociale medier vil kommentarfeltet blive slået fra, så det ikke kan lade sig gøre at kommentere på opslag. På de platforme, der ikke har den funktion (fx LinkedIn), vil eventuelt indhold i kommentarfeltet løbende blive monitoreret og personfølsom information vil blive slettet. Der vil i opslaget blive gjort opmærksom på, at personlig information i kommentarfeltet frabedes.

Det kan således være personalet i de forskellige enheder/hospitaler, der formidler kontakten til de projektansvarlige, såfremt patienten indvilliger heri, men patienten kan også selv tage direkte kontakt til de projektansvarlige.

Det er en forudsætning, at patienten opfylder kriterierne for deltagelse i projektet før projektstart.

Der fremsendes/overleveres skriftlig information om projektet minimum 48 timer før første fremmøde på Psykiatrisk Center København, Forskningsenheden, og der informeres om retten til at medbringe en bisidder.

Ved fremmødet informeres patienten mundtlig om projektet af projektets forskningsassistent og denne svarer på eventuelle spørgsmål før patienten underskriver samtykke erklæringerne. Patienten tilbydes en betænkningstid på 24 timer efter at have modtaget mundtlig og skriftlig information om projektet. Patienter, der selv udtrykker ønske herom, kan samtykke med det samme. Pjecen ”Forsøgspersoners rettigheder i sundhedsvidenskabelige forskningsprojekter” udleveres sammen med deltagerinformationen.

Samtalen vil foregå i et aflukket lokale eventuelt sammen med patientens bisidder. I tilfælde af spørgsmål eller komplikationer vil patienten i hele forsøgsperioden have mulighed for telefonisk at kontakte de forsøgsansvarlige eller andre sundhedsfaglige personer tilknyttet Psykiatrisk Center København, Forskningsenheden.

Under forløbet vil de forsøgsansvarlige have kontakt med patientens kontaktperson i ambulant spiseforstyrrelsesenhed, såfremt patienten samtykker til dette.

## Kontakthyppighed

Ved baseline samt 3 og 6 måneder efter behandlingsafslutning foretages assessment til vurdering af psykopatologi, funktionsniveau samt bivirkninger/negative hændelser.

### Kriterier for diskontinuation

Forsøgsdeltagerne kan når som helt forlade studiet, det er frivilligt at deltage. Hermed specificeret årsager til afbrydelse af studiet:

1. Frivillig afbrydelse af patienten selv; Patienten kan når som helst afbryde deltagelsen i forsøget uden at dette vil påvirke dennes videre behandling i sundhedssystemet.
2. Forkert inklusion af patienten (f.eks. at patienten ikke opfylder inklusions/eksklusionskritererne).

### Procedure for discontinuation

Patienter, som afbryder interventionen under indlæggelse, er fuldt berettiget til dette (jf. frivillighedsprincippet). Patienter, der ønsker at trække sig fra interventionen, vil blive undersøgt forelagt muligheden for at svare på spørgeskema omkring deres tilfredshed med behandlingen, hvis vedkommende ønsker dette.

## Undersøgelser i studiet

### Symptom- og funktionsniveau

Som anført i den engelske forsøgsprotokol, udføres assessments til vurdering af patienternes symptom- og funktionsniveau. Dette undersøges ved hjælp af interviews og selvrapporteringsskemaer og tager ca. 2½ time at gennemføre. Yderligere stilles der mere kvalitative spørgsmål til deltagerne. De kvalitative spørgsmål omhandler deltagernes oplevelse af det at modtage terapi ved brug af virtual reality og hvad, de oplevede var særligt effektivt eller kunne forbedres.

MR-scanninger:

Scanningerne er beskrevet i den engelske forsøgsprotokol ovenfor. Med MR-skanning gives mulighed for at få strukturelle og funktionelle data om hjernens funktion. Scanningerne foretages af trænet personale på Glostrup Hospital og varer maksimalt 90 minutter inkl. pause.

Blodprøver:

Blodprøver tages ved baseline og 3-måneders opfølgning af erfarent personale på blodprøvetagningen på Gentofte Hospital. Yderligere information findes ovenfor i den engelske forsøgsprotokol.

## Behandlingsmodaliteter

Den manualiserede, virtual reality intervention er beskrevet i den engelske forsøgsprotokol.

### Medicinering

Der tilbydes ikke medikamentel behandling som del af dette studium. Patienternes psykofarmakologiske behandling varetages af patientens primærbehandler (ambulant spiseforstyrrelsestilbud i Region Hovedstadens Psykiatri, egen læge eller lign.). Såfremt en projektdeltager er i medikamentel behandling, og ved kontakt med projektet oplyser om bivirkninger hertil, vil man i forskningsprojektet rette henvendelse til patientens primærbehandler.

### Forsøgets bivirkninger, risici og ulemper

Der kan være bivirkninger ved forsøget i form af ubehag ved at bruge virtual reality briller og hovedtelefoner og eventuelt oplevelse af at blive rundtosset eller køresyg, men generelt rapporteres der ikke bivirkninger til virtuel reality behandling^18^. Den afprøvede form for terapi, der er sammenlignelig med såkaldt avatarterapi, har udelukkende vist gavnlig effekt i forhold til at reducere patienternes symptomniveau og deres forpinthed og ubehag forbundet hermed^11,12^.

Yderligere kan der være et mindre ubehag forbundet med blodprøvetagning samt følelse af klaustrofobi ved MR-skanning.

Der forventes derfor ikke at opstå bivirkninger eller negative hændelser som følge af interventionerne i dette studium. Der ses således ingen ulemper ved interventionerne. Skulle der imidlertid opstå bivirkninger eller negative hændelser som følge af terapien, vil det blive registreret og indberettet til Den Videnskabsetiske Komite. Se den engelske forsøgsprotokol for definition af bivirkninger/negative hændelser.

## Databehandling

Projektet anmeldes til Datatilsynet. De forsøgsansvarlige vil indtaste patientens data direkte i en elektronisk CRF (Case Report Form) ved brug af dataindtastningssystemet REDCap. REDCap er et elektronisk dataindtastningssystem, der hostes af CIMT i Region Hovedstaden. REDCap er i overensstemmelse med den danske lovgivning for opbevaring af persondata (Datatilsynet). Data for hver patient er forbundet med et unikt løbenummer. Forsøgsansvarlige og forskningsassistenter er de eneste, der kan tilgå data i REDCap.

Projektet vil blive udført ved overholdelse af databeskyttelsesforordningen og databeskyttelsesloven.

### Journaloplysninger

I forbindelse med henvisning til projektet fra spiseforstyrrelsesenhed på Psykiatrisk Center Ballerup, kan det være nødvendigt at få videregivet oplysninger fra patientens journal med henblik på at identificere om patienten kan indgå i projektet (dvs. opfylder inklusionskriterierne og ikke opfylder eksklusionskriterier). De specifikke helbredsforhold, der indhentes oplysninger om fra journalen, er primært symptomniveau (varighed og omfang af spiseforstyrrelssproblematik, komorbide psykiatriske tilstande, selvmordsrisiko, aktuelle psykofarmakologiske behandling, antal indlæggelser),. Forsøgspersonens samtykke giver den forsøgsansvarlige, sponsor/sponsor repræsentant samt evt. kontrolmyndighed direkte adgang til at indhente relevante oplysninger i patientens journal med henblik på at se oplysninger om forsøgspersonens helbredsforhold, som er nødvendige som led i gennemførelse af forskningsprojekt samt i kontroløjemed, herunder egenkontrol, kvalitetskontrol og monitorering. Alle oplysninger som er indsamlet i projektet og fra journalen vil blive anvendt i projektet i anonymiseret form.

### Erstatningsordning

Forsøget er omfattet af patienterstatningen.

## Videnskabsetisk redegørelse

Projektet vil blive anmeldt til Videnskabsetisk Komité og Videnscenter for Datasikkerhed*.*

Patienter som indgår i projektet, vil blive grundigt informeret både mundtligt og skriftligt, og deltagelse i forsøget accepteres først efter informeret skriftligt samtykke er afgivet. Det vil blive understreget overfor patienterne, at de til enhver tid og uden begrundelse kan trække sig fra projektet, og at dette ikke vil påvirke deres fremtidige behandling i sundhedssystemet.

## Fordelene for patienterne

Patienterne vil gennemgå et udredningsprogram samt have tæt personlig kontakt til de forsøgsansvarlige. Yderligere vil de patienter, der randomiseres til interventionsgruppen, få tilbudt en specialiseret behandling for deres spiseforstyrrelse.

Behandlingen og eventuelle negative hændelser vil blive fulgt tæt. Behandlingen er planlagt således, at den følger gældende kliniske retningslinjer.

Patienterne vil få mulighed for at få tilbagemelding på de undersøgelser, de gennemgår, hvorfor det vil give patienterne, og deres behandlere, yderligere indsigt i patientens individuelle symptomer og vanskeligheder.

## Ulemperne for patienter

Der vurderes primært at være en ulempe forbundet med forsøget i form af muligheden for at blive ”køresyg” ved brug af virtual reality headset. Dette er dog ikke en hyppig bivirkning, og den kan som oftest afsvækkes ved at tilbyde patienten en langsommere indføring i virtual reality scenarierne. Yderligere kan der være en ulempe forbundet med forsøget i form af tidsforbruget ved undersøgelserne (der foretages ved baseline og efter behandlingsafslutning). Tidsforbruget udgør dog kun ca. 2½ undersøgelse pr. undersøgelse.

Yderligere der være et mindre ubehag forbundet med blodprøvetagning samt følelse af klaustrofobi ved MR-skanning.

## Studiets potentiale

Som angivet i engelsk forsøgsprotokol, er spiseforstyrrelse en hyppig lidelse med betydelige omkostninger for individet og samfundsøkonomisk. Der er derfor et åbenlyst behov for at finde effektive og engagerende terapiformer til denne patientgruppe. Dette studium afprøver effekten af en kort, manualiseret, virtual reality-baseret terapiform i et randomiseret, klinisk forsøg. Hvis behandlingen viser sig effektiv og evt. omkostningseffektiv, kan den implementeres i relevante behandlingsenheder under tæt optræning og supervision.

## Økonomiske forhold og klausuler

Forsøget har modtaget støtte i form af bevilling på 2.062.000 kr. fra Danmarks Frie Forskningsråd og en bevilling på 2.224.000 kr fra Region Hovedstadens Psykiatri.

Beløbet skal bruges til aflønning af forsøgsansvarlig, forskningsassistent, psykolog, der udfører behandlingen, samt udvikling af virtual reality behandlingsprogram til at indeholde mad og tilstedeværelse af andre personer.

Virtual reality-programmet er udviklet af den danske, privatejede virksomhed Khora VR, der er specialiseret i VR-løsninger. Khora har ingen indflydelse på studiets design, dataindsamling, analyser eller præsentation af data, ligesom Khora heller ikke har adgang til projektets data. Khora har således ikke habilitetsproblemer i forhold til studiet. Forsøgsansvarlig har ingen økonomisk interesse til Khora.

## Initiativtager

Forskningsleder, specialpsykolog, dr.med, ph.d., lektor Louise Birkedal Glenthøj har taget inititativ til at iværksætte projektet sammen med psykolog Emma Slebsager Ries og psykolog Nina Kappel Hansen fra VIRTU Research Group på Psykiatrisk Center København. VIRTU er i forvejen særdeles aktiv i forskning i virtual reality behandling i psykiatrien.

## Vederlag

Patienter vil ikke modtage nogen godtgørelse, idet de er under behandling. De vil få godtgjort deres transportudgifter og få forplejning på undersøgelsesdagene.

## Offentliggørelse

Såvel positive som negative og inkonklusive forskningsresultater vil blive offentliggjort i internationale tidsskrifter. Resultaterne vil ligeledes blive præsenteret på nationale og internationale møder og kongresser.

Projektet vil blive registreret på [www.clinicaltrials.gov](http://www.clinicaltrials.gov),når der foreligger godkendelse fra Videnskabsetisk Komité samt Videnscenter for Dataanmeldelser, og inden første patient er inkluderet i projektet.

## Organisation

Psykiatrisk Center København stiller kontorlokaler til rådighed. Her vil interviews omkring symptomer, og funktionsniveau foregå og blive udført af forsøgsansvarlige psykologer. Hovedansvaret for behandlingen vil blive varetage af psykolog med specialviden inden for feltet. Projektgruppen består af lektor Louise Birkedal Glenthøj, professor Merete Nordentoft, samt Prof. Nadia Micali fra Psykiatrisk Center Ballerup.

## Gennemførlighed

Forskergruppen har stor erfaring med interventionsforsøg til patienter med psykiatriske lidelser, og der er et godt og tæt samarbejde med ambulatoriet for spiseforstyrrelser på Psykiatrisk Center Ballerup, der gerne henviser patienter til projektet. Spiseforstyrrelsesambulatoriet behandler ca. 1200 patienter hvert år, hvorfor det synes realistisk med inklusion af 62 personer i projektet i studiets 1,5-års inklusionsperiode sammenholdt med yderligere henvisninger fra Landsforeningen mod spiseforstyrrelse (LMS).

# References

1. American Psychiatric Association. *Diagnostic and Statistical Manual of Mental Disorders*. (American Psychiatric Association Publishing, 2022). doi:10.1176/appi.books.9780890425787.

2. Arcelus, J., Mitchell, A. J., Wales, J. & Nielsen, S. Mortality Rates in Patients With Anorexia Nervosa and Other Eating Disorders. *Arch. Gen. Psychiatry* **68**, 724 (2011).

3. Treasure, J. Eating disorders. *Medicine (Baltimore).* **40**, 607–612 (2012).

4. Fichter, M. M. & Quadflieg, N. Mortality in eating disorders - results of a large prospective clinical longitudinal study. *Int. J. Eat. Disord.* **49**, 391–401 (2016).

5. Smink, F. R. E., van Hoeken, D. & Hoek, H. W. Epidemiology of Eating Disorders: Incidence, Prevalence and Mortality Rates. *Curr. Psychiatry Rep.* **14**, 406–414 (2012).

6. Samnaliev, M., Noh, H. L. A., Sonneville, K. R. & Austin, S. B. The economic burden of eating disorders and related mental health comorbidities: An exploratory analysis using the U.S. Medical Expenditures Panel Survey. *Prev. Med. Reports* **2**, 32–34 (2015).

7. NHS National Institute for Health and Clinical Excellence. Eating disorders. NICE clinical guideline. Core interventions in the treatment and management of anorexia nervosa, Bulimia Disorders, nervosa and related eating. *NHS Natl. Inst. Heal. Clin. Excell.* (2004).

8. Yager, J. *et al.* Practice guideline for the treatment of patients with eating disorders third edition. *Am. J. Psychiatry* **163**, 1–128 (2006).

9. Pugh, M. The internal ‘anorexic voice’: a feature or fallacy of eating disorders? *Adv. Eat. Disord.* **4**, 75–83 (2016).

10. Aya, V., Ulusoy, K. & Cardi, V. A systematic review of the ‘eating disorder voice’ experience. *Int. Rev. Psychiatry* **31**, 347–366 (2019).

11. Craig, T. K. *et al.* AVATAR therapy for auditory verbal hallucinations in people with psychosis: a single-blind, randomised controlled trial. *The Lancet Psychiatry* **5**, 31–40 (2018).

12. du Sert, O. P. *et al.* Virtual reality therapy for refractory auditory verbal hallucinations in schizophrenia: A pilot clinical trial. *Schizophr. Res.* (2018) doi:10.1016/j.schres.2018.02.031.

13. Smith Lisa Charlotte, Mariegaard, Lise, Vernal, Ditte Lammer, Christensen Annette Gosvig, Albert, Nikolai, Thomas, Neil, Hjorthøj, Carsten, Glenthøj, Louise Birkedal, Nordentoft, M. The CHALLENGE-trial: the effects of a virtual reality-assisted exposure therapy for persistent auditory hallucinations versus supportive counselling in people with psychosis: study protocol for a randomised clinical trial. *Under Rev.* (2022).

14. Rus-Calafell, M. *et al.* The Role of Sense of Voice Presence and Anxiety Reduction in AVATAR Therapy. *J. Clin. Med.* **9**, 2748 (2020).

15. Frank, G. K. W. Advances from neuroimaging studies in eating disorders. *CNS Spectr.* **20**, 391–400 (2015).

16. Scharner, S. & Stengel, A. Alterations of brain structure and functions in anorexia nervosa. *Clin. Nutr. Exp.* **28**, 22–32 (2019).

17. Celeghin, A. *et al.* Brain Correlates of Eating Disorders in Response to Food Visual Stimuli: A Systematic Narrative Review of FMRI Studies. *Brain Sci.* **13**, 465 (2023).

18. Rus-Calafell, M., Garety, P., Sason, E., Craig, T. J. K. K. & Valmaggia, L. R. Virtual reality in the assessment and treatment of psychosis: a systematic review of its utility, acceptability and effectiveness. *Psychol. Med.* **48**, 362–391 (2018).

19. Pot-kolder, R. M. C. A. *et al.* Articles Virtual-reality-based cognitive behavioural therapy versus waiting list control for paranoid ideation and social avoidance in patients with psychotic disorders : a single-blind randomised controlled trial. **0366**, (2018).

20. Garner, D. M., Olmstead, M. P. & Polivy, J. Development and validation of a multidimensional eating disorder inventory for anorexia nervosa and bulimia. *Int. J. Eat. Disord.* **2**, 15–34 (1983).

21. Haddock, G., McCarron, J., Tarrier, N. & Faragher, E. B. Scales to measure dimensions of hallucinations and delusions: the psychotic symptom rating scales (PSYRATS). *Psychol. Med.* **29**, 879–89 (1999).

22. Cooper, P. J., Taylor, M. J., Cooper, Z. & Fairbum, C. G. The development and validation of the body shape questionnaire. *Int. J. Eat. Disord.* **6**, 485–494 (1987).

23. Thabrew, H., Stasiak, K., Bavin, L. M., Frampton, C. & Merry, S. Validation of the Mood and Feelings Questionnaire (MFQ) and Short Mood and Feelings Questionnaire (SMFQ) in New Zealand help-seeking adolescents. *Int. J. Methods Psychiatr. Res.* **27**, 1–9 (2018).

24. Neff, K. D. *et al.* Development and Validation of the Self-Compassion Scale for Youth. *J. Pers. Assess.* **103**, 92–105 (2021).

25. Rabin, R. & De Charro, F. EQ-5D: A measure of health status from the EuroQol Group. *Ann. Med.* **33**, 337–343 (2001).

26. Larsen, D. L., Attkisson, C. C., Hargreaves, W. a. & Nguyen, T. D. Assessment of client/patient satisfaction: development of a general scale. *Eval. Program Plann.* **2**, 197–207 (1979).

27. Kennedy, R. S., Fowlkes, J. E., Berbaum, K. S. & Lilienthal, M. G. Use of a motion sickness history questionnaire for prediction of simulator sickness. *Aviat. Space. Environ. Med.* **63**, 588–93 (1992).

28. Luszczynska, A., Scholz, U. & Schwarzer, R. The general self-efficacy scale: Multicultural validation studies. *J. Psychol. Interdiscip. Appl.* **139**, 439–457 (2005).

29. Zigmond, A. S. & Snaith, R. P. The Hospital Anxiety and Depression Scale. *Acta Psychiatr. Scand.* **67**, 361–370 (1983).

30. Stanghellini, G., Castellini, G., Brogna, P., Faravelli, C. & Ricca, V. Identity and Eating Disorders (IDEA): A Questionnaire Evaluating Identity and Embodiment in Eating Disorder Patients. *Psychopathology* **45**, 147–158 (2012).

31. Gioia, G. A., Isquith, P. K., Retzlaff, P. D. & Espy, K. A. Confirmatory Factor Analysis of the Behavior Rating Inventory of Executive Function (BRIEF) in a Clinical Sample. *Child Neuropsychol.* **8**, 249–257 (2003).

32. Rouel, M., Raman, J., Hay, P. & Smith, E. Validation of the Behaviour Rating Inventory of Executive Function – Adult Version (BRIEF-A) in the obese with and without binge eating disorder. *Eat. Behav.* **23**, 58–65 (2016).

33. Witmer BG, S. M. Measuring Presence in Virtual Environments: A Presence Questionnaire. *Presence Teleoperators Virutal Environ.* **7**, 225–240 (1998).

34. Bernstein DP, Fink L, Handelsman L, et al. Initial reliability and validity of a new retrospective measure of child abuse and neglect. *Am. J. Psychiatry* **151**, 1132–1136 (1994).

35. Weissman, A.N, & Beck, A. T. Development and validation of the Dysfunctional Attitude Scale: a preliminary investigation. (1978).

36. Freeman, D. *et al.* Virtual reality in the assessment, understanding, and treatment of mental health disorders. *Psychol. Med.* **47**, 2393–2400 (2017).

37. Geraets, C. N. W., van der Stouwe, E. C. D., Pot-Kolder, R. & Veling, W. Advances in immersive virtual reality interventions for mental disorders: A new reality? *Curr. Opin. Psychol.* **41**, 40–45 (2021).

38. Pot-Kolder, R. *et al.* Cost-effectiveness of virtual reality cognitive behavioral therapy for psychosis: Health-economic evaluation within a randomized controlled trial. *J. Med. Internet Res.* **22**, 1–13 (2020).
