# Supplementary material for: The Dialogue study: Protocol for a randomized clinical trial evaluating the efficacy of virtual reality-based psychotherapy plus treatment as usual versus treatment as usual for eating disorders
Source: PLoS One. 2025 Jul 28;20(7):e0319875. doi: 10.1371/journal.pone.0319875 (PMC12303276; doi:10.1371/journal.pone.0319875)
Supplement: S1 Fig — Schedule showing the timing of participant enrolment, interventions, and assessments. Includes all key study procedures and follow-up timepoints. (DOC) [file pone.0319875.s003.doc]

S1 Fig: Participant timeline

Assessed for eligibility

**Enrollment**

Excluded

- Not meeting inclusion criteria
- Meeting exclusion criteria
- Refused to participate
- Other reasons

Included

**Allocation**

Baseline assessment (T= 0)

Randomisation

#

**Intervetionn**

**Follow-up**

**Analysis**

**Treatment as usual** (TAU)

(n = 48)

**VR-based avatar intervention** (7 individual sessions)

+

Treatment as usual (TAU)

(n = 48)

3 months post baseline

6 months post baseline

3 months post baseline

6 months post baseline

Intention-to-treat-analysis

Intention-to-treat-analysis
