# Supplementary material for: The Dialogue study: Protocol for a randomized clinical trial evaluating the efficacy of virtual reality-based psychotherapy plus treatment as usual versus treatment as usual for eating disorders
Source: PLoS One. 2025 Jul 28;20(7):e0319875. doi: 10.1371/journal.pone.0319875 (PMC12303276; doi:10.1371/journal.pone.0319875)
Supplement: Attachment — Submitted filename: Response to Reviewers.docx [file pone.0319875.s005.docx]

**Dear editor,**

Thank you for reviewing our manuscript and for the invitation to submit a revised version. We have carefully addressed all the points raised during the editorial process and by the reviewers. We are grateful for their time and thoughtful feedback, which has helped us significantly strengthen the manuscript.

Kind regards,

Louise Birkedal Glenthøj, corresponding author.

We have corrected the following editorial and formatting comments, to align with PLOS ONE’s requirements.

**Comments from the Editor:**

1. *Please ensure that your manuscript meets PLOS ONE’s style requirements, including those for file naming.*
2. *We note that the grant information you provided in the ‘Funding information’ and ‘Financial disclosure’ sections do not match. When you resubmit, please ensure that you provide the correct grant numbers for the rewards you received for your study in the ‘Funding information’ section.*
3. *Your ethics statement should only appear in the Methods section of your manuscript. If your ethics statement is written in any section besides the Methods, please move it to the Methods section and delete it from any other section. Please ensure that your ethics statement is included in your manuscript, as the ethics statement entered into the online submission form will not be published alongside your manuscript.*
4. *Please provide a complete Data Availability Statement in the submission form, ensuring you include all necessary access information or a reason for why you are unable to make your data freely accessible. If your research concerns only data provided within your submission, please write "All data are in the manuscript and/or supporting information files" as your Data Availability Statement.*
5. *Please ensure that you refer to Figure 2 in your text as, if accepted, production will need this reference to link the reader to the figure.*
6. *Please remove all personal information, ensure that the data shared are in accordance with participant consent, and re-upload a fully anonymized data set.*

*Note: spreadsheet columns with personal information must be removed and not hidden as all hidden columns will appear in the published file.*

*Additional guidance on preparing raw data for publication can be found in our Data Policy (https://journals.plos.org/plosone/s/data-availability#loc-human-research-participant-data-and-other-sensitive-data) and in the following article:* [*http://www.bmj.com/content/340/bmj.c181.long*](http://www.bmj.com/content/340/bmj.c181.long)*.*

7. *Please include captions for your Supporting Information files at the end of your manuscript, and update any in-text citations to match accordingly. Please see our Supporting Information guidelines for more information:* [*http://journals.plos.org/plosone/s/supporting-information*](http://journals.plos.org/plosone/s/supporting-information)*.*

**Below we outline our responses to the reviewer’s comments:**

**Reviewer one:**

*This is a RCT interesting study assessing the effectiveness of a VR intervention to reducing eating disorder symptoms.*

**Response:**

Thank you for thoughtful evaluation of our study and for finding it interesting.

*Some minor comments:*

1. *In Line 153 or in section 508 - can the authors note the allocation ratio between Intervention: TAU.*

**Response:**

Thank you for this comment. We agree, the allocation ratio should be stated clearly.

The following paragraph has been added to the ‘Trial design’ section:

“Participants will be randomly assigned to the intervention plus TAU or TAU in a 1:1 ratio (48 participants in each group*)*.”

2. *What’s the rationale for not also stratifying by eating disorder symptom severity at baseline incase by chance there is imbalance between groups and is this prognostic of the outcome? i.e eht EDE-Q?*

**Response:**

Thank you for raising this important point. We did consider stratifying by baseline eating disorder symptoms, however, we estimated that this would add another layer of complexity to the already difficult task of recruiting. Additionally, our randomization scheme already stratifies by site. Adding an additional stratification variable would increase the likelihood of half-empty randomization blocks, in effect resulting in imbalances within strata. These two factors led us to drop the stratification by severity.

3. *Line 154- define ITT - as you have done in line 612 - then no need to repeat in 612 or remove ITT sentence in Line 154.*

**Response:**

Thank you for this observation. The ITT sentence has been deleted in line 154.

4. *When patients withdraw/discontinue for whatever reason - does the consent also allow for the researchers to use the data up to the point of withdrawal - this should be made explicit in the protocol?, as it says they can withdraw consent - but not clear when it comes to other aspects such data sharing with third parties.*

**Response:**

Thank you for this helpful comment. We agree that this should be clearly stated in the protocol. Participants are informed during the consent process that they may withdraw from the study at any time without consequence (e.g., drop-out). The participants will be asked if they wish to have their data deleted upon withdrawal. If they do not request for their data to be deleted, data collected up to the point of withdrawal will be retained and used in analyses, in line with ethical guidelines and national data protection regulations. If data remain linkable (e.g., via a pseudonym or code), it may be deleted upon request prior to publication. However, once data are fully anonymized or shared in de-identified form with third parties, individual data can no longer be withdrawn.

We have clarified this in the ethics section that now explicitly states:

‘Participants will be asked whether they wish to have their data deleted upon withdrawal. If they do not request deletion, data collected up to the point of withdrawal will be retained and used in analyses, in accordance with ethical guidelines and national data protection regulations. If data remain linkable (e.g., via a pseudonym or code), it may be deleted upon request prior to publication. However, once data are fully anonymized or shared in de-identified form with third parties, individual data can no longer be withdrawn.’

5. *In the sample size section- can you state the attrition %age as it looks to be aroung 15%.*

**Response:**

We agree that it would be helpful to add the attrition %age. An argument for this percentage has also been added:

The following has been added to the sample size section:

‘Attrition rates in RCT’s investigating psychological treatment for eating disorders has been reported to range from 0 to 34.2% for studies with immediate follow-up and 2.4% to 21.4% for studies with three-month post intervention follow-up ^61^. To account for potential attrition and deviations from the assumptions, we have increased the total sample size by 20% to 96 participants.’

6. *In the statistical methods sections add* *reporting guidelines will follow CONSORT guidelines. Also need a few sentence* *aroung planed descriptive sumaasries of baseline characteristis, i.e by randomised group.*

**Response:**

Thank you for these helpful suggestions. It has been added to the manuscript that reporting will follow CONSORT guidelines, see ‘Statistical analysis’ section.

We have also added a description of the planned descriptive summaries of baseline characteristics. Specifically, we will present baseline demographic and clinical characteristics using descriptive statistics (e.g., means, standard deviations, medians, and proportions) for each group. This will allow us to assess comparability between groups at baseline.

These details have been added to the Statistical Analysis section:

‘‘Baseline demographic and clinical characteristics will be summarized for each group, using descriptive statistics (e.g., means, standard deviations, medians, and range for continuous variables, frequencies and proportions for categorical) to assess comparability between groups at baseline.’

7. *As this an open label study - was the SAP signed off prior to recruitment see ICH guidelines on this.*

**Response:**

Thank you for this important question. The statistical methods, as they are outlined in this manuscript, was signed off before recruitment and was uploaded to clinical trials within 21 days of enrollment of the first participant, in compliance with section 801 of the Food and Drug Administration Amendments Act (FDAAA 801). A more detailed SAP is currently in preparation and will be made publicly available before database lock (i.e., before the dataset is finalized and analysis starts).

8. *Have some brief mention of the planned primary analysis and that treatment effect will be reported alongside 95%CI and associated p-value, i.e 5% as the significance level. i.e mention of missing data- can you state which outcomes this relates to, since primary outcome is collected at a single time point and baseline (which I assume will be adjusted for in the models).*

**Response:**

Thank you for this helpful comment. We agree that it should be stated that effect will be reported alongside 95%CI and associated p-value, i.e. 5% as the significance level.

The planned primary analysis in the statistical methods section now reads as follows:

‘Analyses will be performed on the ITT population for the primary and secondary endpoints, i.e., analyzing all randomized participants according to the arm they were randomized to. The analysis method will be ANCOVA of change from baseline with treatment as factor and baseline value as covariate at the corresponding post-baseline visit, i.e., month 12. Treatment difference point estimates will be reported together with 95% CIs and p-value. Missing values will be multiply imputed. For the exploratory endpoints, a modified ITT set will be used, requiring at least one post-baseline measurement for the inclusion in the analysis for each endpoint. The analysis method will be linear mixed effects model. Results will be reported for both post-baseline visits. Details of model specifications, imputation methods & assumptions, sensitivity analyses and subgroup analyses will be provided in the statistical analysis plan (SAP), that will be made publicly available prior to data lock (i.e., before the dataset is finalized and analysis starts).’

The variable for the primary endpoint is collected at two post-baseline visits, i.e., month 12 and 24, but the primary endpoint is set at month 12. Any missingness in this outcome at month 12 for the primary analysis will be addressed using multiple imputation. That is, for the parameters of primary and secondary endpoints, which are evaluated at month 12, multiple imputation will be used. For the exploratory endpoints, missing data will be indirectly handled through mixed effects models.

These additions have been made in the Statistical Analysis section:

‘For the primary and secondary endpoints, missing data will be multiply imputed under MAR. Sensitivity analyses will involve exploring departures from the MAR assumption, i.e., a tipping point analysis is planned to assess how far must the imputed values deviate from the MAR assumption before the conclusion of the primary analysis is overturned.

For the exploratory endpoints, missing data will be implicitly handled by the linear mixed effects model.’

**Reviewer 2:**

*The study is using AVATAR therapy, originally designed for psychotic disorders, to treat eating disorders. However, it does not introduce a completely new method and needs to justify why this approach would be effective.*

**Response:**

Thank you for this valuable comment. While AVATAR therapy itself is an established intervention, its application to individuals with eating disorders, in a fully immersive format, represents a novel and clinically meaningful adaptation. Many individuals with an eating disorder report experiencing a persistent, critical, and controlling inner voice, that may reinforce disordered behaviors and impedes recovery. Despite its clinical relevance, this inner voice is rarely targeted directly in standard treatments. AVATAR therapy offers a structured and experiential way to externalize and interact with this voice offering a new pathway for therapeutic intervention. Externalization is particularly important in the context of eating disorders, which are often ego-syntonic and characterized by ambivalence towards change. By giving form and voice to the internal critical dialogue, AVATAR therapy may facilitate a clearer separation between the individual and the disorder. This may support the development of a stronger, recovery-oriented self-narrative; something that traditional talk therapies may struggle to achieve with the same immediacy or emotional impact. Thus, while the therapeutic modality is not entirely new, its application in this context is innovative and addresses a critical and underexplored mechanism in eating disorder pathology. We believe this adaptation holds promise for enhancing treatment outcomes and expanding the clinical utility of AVATAR therapy.

In the ‘background’ section of the manuscript, we have stressed the innovativeness of applying a fully immersive avatar intervention to eating disorders:

‘The experience of a more powerful eating disorder voice is associated with increased use of compensatory behaviors (e.g., fasting, vomiting, laxative misuse, compensatory exercise) and longer illness duration ^20^. In line with this, research suggests that the individual’s subordinate position to the eating disorder voice could play a substantial role in maintaining eating disorder symptoms and contribute to challenges in treatment engagement ^20^ Despite its clinical relevance, this power dynamic is rarely addressed directly in standard treatments. Targeting it in therapy may enhance clinical outcomes and treatment engagement by helping patients regain control over the voice and strengthen their identity beyond the disorder.’

Plus:

‘The first AVATAR Therapy for psychosis and anorexia nervosa was delivered on a 2D computer screen. Our research group recently tested the efficacy an immersive 3D avatar intervention for psychosis, mirroring the positive findings from the original AVATAR therapy trial^27^. Building on this, we adapted the virtual reality (VR) software to individuals with eating disorders. In a feasibility study (n=10), this immersive version met the predefined thresholds for feasibility and acceptability and showed promising preliminary treatment effect (unpublished work). Based on these initial findings, the current study aims to investigate the efficacy of an immersive VR-based avatar intervention for eating disorders. The study represents the first fully powered, large-scale, methodologically rigorous clinical trial to explore whether a relational, dialogue-based approach, such as avatar intervention can reduce symptoms and improve quality of life for patients with an eating disorder. The application of avatar-based intervention to eating disorders is an innovative approach that addresses a critical and underexplored mechanism in eating disorder pathology. By giving form and voice to the internal critical dialogue, avatar-based intervention may facilitate a clearer separation between the individual and the disorder, an important mechanism in the context of eating disorders which are often ego-syntonic and characterized by ambivalence towards change ^21,28^. The immersive format of VR-based avatar intervention may support the development of a stronger, recovery-oriented self-narrative; something that traditional talking therapies may struggle to achieve with the same emotional impact. If the study yields positive findings, it could pave the way for new research on psychological interventions for eating disorders. The next step would involve replicating these results across diverse settings.’

1. *It assumes that the "eating disorder voice" is similar to voices in psychotic disorders, but this connection is not clearly proven. More evidence is needed to confirm that AVATAR therapy will work in the same way.*

**Response:**

Thank you for highlighting this important point regarding the scientific rationale for the intervention. While there is no evidence that the “eating disorder voice” is identical to the voice experience in psychotic disorders in all respects, research does suggest that the two phenomena share a key relational feature: the subordinate position that individuals often adopt in relation to the voice. This overlap has sparked interest in applying relational dialogue-based interventions for voice hearing to target the eating disorder voice. This approach has been supported by emerging qualitative findings (see Cardi et al., Hibbs et al., and Ling et al.). As a relational therapy, avatar intervention specifically targets this shared dynamic, which provides a compelling rationale for testing its applicability in eating disorder populations who exhibit the same relational pattern.

While the empirical foundation is still developing, we believe that exploring well-theorized, transdiagnostic mechanisms such as this relational dynamic is essential to advancing clinical innovation.

We have revised the manuscript to clarify the conceptual link between voice-hearing in psychosis and the eating disorder voice, and to better highlight the emerging evidence supporting this therapeutic adaptation:

‘A growing body of research suggests that the experience of an inner eating disorder voice shares a key relational feature with distressing verbal auditory hallucinations, namely the subordinate position that individuals often adopt in relation to the voice ^20–23^. This overlap has prompted interest in applying relational therapies developed for voice-hearing (e.g. voice dialogue), to address the eating disorder voice, an approach supported by emerging qualitative findings ^22,23^AVATAR Therapy, a novel relational intervention, originally designed for voice hearing targets this power imbalance. In the therapy, participants engage in real-time dialogue with a digital avatar representing their distressing voice, allowing them to challenge this power dynamic. The intervention has demonstrated efficacy in reducing the severity of the psychotic voice and alleviating associated distress within two fully powered randomized controlled trials^24,25^.Given that AVATAR Therapy directly targets the shared relational dynamic between voice hearers and people with eating disorders, it provides a compelling rationale for testing the intervention’s applicability in eating disorder populations^18,26^.’

2. *The research is based on a small study using a simple (2D) version of the therapy, which is not enough proof to justify testing a more advanced (3D) version in a large trial. More small-scale studies should be done first.*

**Response:**

Thank you for this important methodological point. Before setting up the RCT, we conducted a feasibility study to test the 3D version of the intervention (n=10), highlighting descriptive statistics on feasibility as well as qualitative data, informing the treatment protocol of the RCT, and exploring acceptability. We also included a preliminary analysis of treatment effect. The study found that the 3D intervention was feasible and acceptable for individuals with eating disorders and showed preliminary effect on eating disorder symptoms, among other outcomes. A manuscript detailing these findings is currently in preparation and therefore not included in the protocol manuscript at first.

In the ‘introduction’ we have added a brief mention of our feasibility study:

‘Our research group recently tested an immersive avatar intervention for psychosis, mirroring the positive findings from the original AVATAR therapy trial^27^. Building on this, we adapted the virtual reality (VR) to individuals with eating disorders. In a feasibility study (n=10), the intervention met the predefined thresholds for feasibility and acceptability and showed promising preliminary treatment effect (unpublished work). Based on these initial findings, the current study aims to investigate the efficacy of this VR-based avatar intervention for eating disorders.’

3. *Virtual reality therapy requires costly equipment and training. The study does not explain how this will be managed in hospitals and clinics, making it difficult to implement widely.*

**Response:**

Thank you for this important observation. We agree that implementation considerations, including equipment costs and staff training, are crucial for the broader use of VR-based therapies. While the primary focus of this trial is to evaluate efficacy under controlled conditions, we acknowledge the importance of considering real-world applicability and have now clarified this distinction more explicitly in the revised manuscript. To avoid any potential confusion regarding the study’s aims, we have removed references to cost-effectiveness from the protocol, as this will not be assessed in the current trial. Additionally, the manuscript title has been updated to reflect the emphasis on **efficacy** rather than **effectiveness**.

In the title of the manuscript, we have replaced the word “effectiveness” with efficacy:

‘The Dialogue Study: Protocol for a randomized clinical trial evaluating the efficacy of virtual reality-based psychotherapy plus treatment as usual versus treatment as usual for eating disorders.’

That said, we have considered implementation in a preliminary way. VR technology is becoming increasingly accessible and cost-effective as noted by Freeman et al. (2017). While training clinicians in VR-based interventions would entail additional investment, we envision this treatment as a specialized adjunct to standard care, likely to be delivered within specialist units for eating disorders rather than in general clinical settings. This focused model of deployment could help manage both cost and training needs in the long term. We hope this clarification addresses the concern, while also acknowledging the appropriate timing for implementation considerations within the research pipeline.

4. *The study does not discuss how long the benefits of the treatment will last. It should mention whether patients might return to old habits over time and how to prevent this.*

**Response:**

Thank you for highlighting this important consideration. Our study includes a 3-month post-intervention follow-up, during which we will assess the durability of treatment effects. While this timeframe provides some insight into short-term maintenance, we recognize that longer-term outcomes are also important and may require future investigation.

As long-term follow-up is not included in the current study, discussion of long-term effects has been added in the section on limitations of the study:

‘Although VR-based interventions have demonstrated lasting effects in related fields, such as anxiety disorders ^73^, the current study does not include extended follow-up periods. As such, it cannot address the durability of therapeutic gains over time. Future research should incorporate long-term follow-up to evaluate the persistence of this VR-based treatment’s effect.’

To support the maintenance of treatment effects beyond the intervention period, patients continue to receive standard care before and after the VR-based intervention. In addition, the treatment protocol includes audio recordings designed to reinforce key therapeutic elements. These recordings can be used by patients after the intervention ends to help sustain progress and potentially reduce the risk of relapse, although the potential of sustained effects will not be formally investigated.

In the section ‘Provisions for post-trial care’ it is stated that:

**‘**Participants will receive their standard care before, during and after trial intervention.’

In the manuscript’s ‘intervention description’ section we have clarified how the intervention seeks to sustain progress and address risk of relapse (see text highlighted in green):

‘As part of the intervention, participants are encouraged to take a picture of the avatar and are given audio recordings of the avatar-dialogues. They are then asked to listen to the recordings between sessions to further bolster their sense of control/autonomy and strengthen motivation for change. In addition, these recordings can be used by patients post-intervention to help sustain progress and potentially reduce the risk of relapse.’.

5. *There are not enough details about how the study will measure success. It should clearly define what changes in patients' thinking or behavior will be used to evaluate the therapy’s effectiveness.*

**Response:**

Thank you for pointing out that the criteria for success should be stated more clearly. As this study evaluates the VR-intervention’s efficacy, success is defined by a clinical meaningful difference between the groups on our primary outcome of eating disorder symptoms, measured by the EDE-Q global scale. With regard to secondary outcomes we are measuring improvements in specific cognitive and behavioral domains relevant to eating the disorder voice experience and eating disorder psychopathology, such as voice appraisal, identification with the eating disorder, emotion regulation and motivation for change.

We have rephrased our study objectives in the manuscript, to state this more clearly:

**‘Primary Objective:** To evaluate whether a VR-based avatar intervention combined with treatment as usual (TAU) results in greater reductions in eating disorder symptoms compared to TAU alone, as measured by the global score on the Eating Disorder Examination Questionnaire (EDE-Q). A clinically meaningful difference is defined as a between-group difference of ≥0.83 on the EDE-Q global score.

**Secondary objectives**: To evaluate whether the VR-based avatar intervention plus treatment as usual (TAU), compared to TAU alone, leads to greater improvements in cognitive and behavioral domains associated with the eating disorder voice experience and eating disorder psychopathology. These domains include: Voice appraisal (e.g., perceived power, omnipotence, and malevolence of the eating disorder voice), identification with the eating disorder, level of depressive symptoms, and motivation for change (e.g., readiness and confidence to recover). Each domain will be assessed using validated self-report measures, and between-group differences will be evaluated at post-intervention.

**Exploratory objectives**: To examine the effects of the VR-based avatar intervention plus TAU on a broader range of cognitive, affective, behavioral outcomes related to eating disorder psychopathology. These include measures of body image, self-compassion, emotion regulation and cognitive flexibility. Full details of all exploratory outcomes, including timing and measures, are provided in the section ‘Exploratory Outcomes’.

1. *Talking to an avatar of their eating disorder voice could be emotionally distressing for some patients. The study does not discuss possible negative effects or how they will be handled.*

**Response:**

Thank you for raising this important point. We fully agree that the intervention can be emotionally triggering, making it important to discuss negative effects and how they will be handled during the trial. We have made the following changes to the manuscript to detail how negative effects will be addressed in the trial: 1) We have added information on how trial therapists handle emotional distress and negative effects 2), we have added a section in the discussion concerning ethical implications related to the therapy:

1)The trial therapists have a procedure for handling emotional distress. We have added this procedure to the section ‘Adverse event reporting and harms’ (new text highlighted in green):

‘The main known side-effect to VR-based interventions is cyber sickness, resembling common motion sickness. If encountered, it typically resolves after a few VR-sessions as tolerance develops. VR-based psychotherapy is generally well tolerated 65. Hence, we do not expect any serious adverse events to occur. As this is an emotion focused intervention set in an immersive condition, we do expect that participants may experience distress as a temporary part of the therapeutic process, especially in the first sessions. Participants will be informed of this enabling them to make informed decisions regarding participation and adherence, and to prepare them for managing any potential transitory reactions 66. As detailed in the section 'Intervention description,' the therapists will monitor the participants' levels of distress throughout the therapy course, adjusting the intervention accordingly.

If a participant becomes overwhelmed, therapists will assist with in-session and at-home calming strategies and follow up the next day by phone or text. Participants will also continue receiving standard care before, during, and after the intervention to support safety. Any acute events impacting emotional or physical health will initiate immediate action, including contacting the participant’s primary clinician, notifying relatives, or escorting the participant to acute psychiatric care, as appropriate. The following serious adverse advents are monitored throughout the study: 1) hospital admissions; 2) suicide attempts; 3) violent incidents necessitating police involvement (whether victim or accused); 4) self-harm; 5) deaths.’

We have also stressed that the ethics committee overseeing the trial, have the authority to pausing or terminating the trial:

‘The Danish Health Research Ethics Committee have the authority to recommend pausing or terminating the trial.’

The following section on ethical implications of the intervention has been added to the discussion:

‘**Ethical implications:**

Avatar-based intervention for eating disorders may be emotionally demanding, as it involves engaging participants in dialogues with digital a representation of their often abusive eating disorder voice. While many participants in avatar-based therapy report empowerment and emotional relief, the approach carries ethical risks, particularly when therapists voice abusive or discriminatory content through the avatar ^68^. As a result, patient safety has been prioritized both in the conduct of the trial and in the evaluation of the intervention. Trial therapists are required to assess each participant’s psychological readiness, monitor for distress, ensure ongoing informed consent, and actively manage any negative effects, as detailed in the ‘Adverse Events Reporting and Harms section’. In addition, the study will systematically assess participant reported negative outcomes, using the Negative Effects Questionnaire (NEQ).’

**Reviewer 3:**

*Thank you for the opportunity to review your protocol investigating the effect of a virtual reality-based intervention for eating disorders titled “The Dialogue Study: A randomized clinical trial evaluating the effectiveness of a virtual reality-based intervention plus treatment as usual versus treatment as usual for eating disorders”.*

*Your proposed protocol presents a novel and potentially impactful approach to addressing eating disorder pathology through VR-based avatar interventions. What follows are some suggested changes you may want to consider incorporating in outlining the reasoning for the trial and how you plan to address some of common challenges of RCTs in the analysis and reporting.*

**Response:**

Thank you for your positive feedback regarding the study’s aim and your helpful suggestions and references to improve the manuscript.

1. Introduction:

*The economic burden of eating disorders could be supported with specific data or references (e.g., Schmidt et al., 2016). Similarly, further evidence on the role of the eating disorder voice in symptom maintenance and treatment resistance could be included (e.g., Aya et al., 2019)*

**Response:**

We agree that supporting the economic burden of eating disorders with specific data and references will strengthen the rationale and provide important context for the significance of the intervention.

The paragraph on the economic burden of eating disorders now reads:

‘Additionally, eating disorders are associated with a significant economic burden, including health care costs, reduced employment rates, and loss of productivity. In the European union the annual costs approaches €1 trillion, exceeding the economic impact of anxiety and depression7.’

As also proposed, we have further stressed the evidence on the eating disorder voice’s role in in symptom maintenance and treatment resistance, by applying your suggested reference:

‘The experience of a more powerful eating disorder voice is associated with increased use of compensatory behaviors (e.g., fasting, vomiting, laxative misuse, compensatory exercise) and longer illness duration ^20^. In line with this, research suggests that the individual’s subordinate position to the eating disorder voice could play a substantial role in maintaining eating disorder symptoms and contribute to challenges in treatment engagement ^20^ Despite its clinical relevance, this power dynamic is rarely addressed directly in standard treatments. Targeting it in therapy may enhance clinical outcomes and treatment engagement by helping patients regain control over the voice and strengthen their identity beyond the disorder ^17,22–24^.’

2. *Intervention Design and Mechanisms of Change:*

*The explanation of embodied externalization and dialogue as mechanisms of change is a key strength of your manuscript. The comparison to chair work techniques is particularly insightful, as it situates your approach within existing therapeutic frameworks while highlighting the advantages of VR immersion. You may wish to further discuss how factors such as presence, emotional engagement, and therapeutic alliance in the VR setting compares to more traditional interventions (e.g., Natali et al., 2023; Ward et al 2020). In addition to the discussing the potential long-term benefits of avatar-based interventions (e.g., Freeman et al., 2017).*

**Response:**

Thank you for suggesting adding these relevant points to nuance the discussion further.

We have incorporated points on therapeutic alliance, emotional engagement and presence compared to traditional talking therapy to the discussion, that now reads as follow (new additions highlighted in green):

‘**Embodied externalization and dialogue as an important mechanism of change**

Prominent interventions for eating disorders such as Cognitive-Behavioral Therapy, the Maudsley Model of Anorexia Nervosa Treatment for Adults, and Focal Psychodynamic Therapy, typically focus on challenging the ‘irrationality’ and ‘unhelpfulness’ of thought content 22. However, these traditional approaches often overlook the patient’s relationship with these thoughts 22. In contrast, avatar-based therapy, incorporates therapeutic modalities such as relational and narrative therapies, which emphasize addressing the subordinate stance that patients often adopt towards these problematic thoughts or parts of the self. As evident within the frame-work of avatar-based therapy, relational approaches often utilize externalization techniques to enable patients to challenge the influence of these thoughts or parts of self by facilitating a dialogue with them 22,67.

The application of avatars or computer-generated representations expands upon techniques like the chair work method, a dialogue-based approach that externalizes a part of the self in a physical form and space. 22,67. The chair work technique involves using two chairs to represent different aspects of the self, with participants physically shifting between chairs to adopt the perspectives of each part in real time 23,24,67. These embodied approaches, has been shown to be particularly useful for patients, who, like those with eating disorders, often over-identify with a specific part of the self, enhancing their ability to differentiate themselves from their inner experiences 22,67.

However, the chair work method still depends heavily on the participants’ imagination to embody the different parts of the self. In contrast, employing avatars allows patients to engage in a dialogue with a part of self, as if it were a*n external independent* entity. Findings in cognitive science, suggest that experiencing oneself acting on an external entity, rather than on an internal representation, enhances the feeling of agency (e.g., the ability to make independent choices and act on them) ^67^. Building on this, avatars not only help patients differentiate themselves from their inner experiences but also promote a sense of autonomy, potentially offering a more effective position to create dissonance between the self and the eating disorder and achieve greater sense of control. Compared to traditional talking therapies avatar-based interventions also poses new possibilities and challenges in the therapeutic relationship. When the therapist voices the eating disorder’s often abusive content, it requires considerable training in the method to ensure the dialogue remains therapeutically contained and it poses new ethical concerns (also see discussion on ‘Ethical implications’) ^68^. On the other hand, patients with an eating disorder often show ambivalence towards recovery, rendering motivational work a central part of treatment. Avatar-based intervention may help mitigate a common difficulty in this treatment phase: the tendency for therapists to argue for change, unintentionally positioning patients to defend the eating disorder ^69,70^. By externalizing the eating disorder through an avatar, the patient can respond to the disorder directly, from other parts of the self, replacing the oppositional dynamic between therapist and patient with internal dialogue.

**The potential of VR enhanced externalization**

Unlike traditional talking therapies, which relies on reflective insight, avatar-based interventions provide an experiential context where individuals engage directly with an embodied part of the self. This immersive format allows for real-time work on “hot” cognitive, emotional, and relational processes ^68^. A newly published study on AVATAR therapy for psychosis found that sense of presence in the avatar dialogue was associated with emotions such as control and that higher levels of presence were linked to greater reductions in auditory verbal hallucinations, suggesting that presence may be a key component of therapeutic outcomes in avatar-based therapy ^71^. The current study represents a pioneering approach within externalization techniques, employing 3D immersive technology to embody the eating disorder part of self. Virtual reality enables users to interact within a simulated environment as if they were physically present in that environment ^67^, making it possible for participants to experience a sense of being present with their eating disorder voice in the same physical space ^72^. This heightened sense of presence may offer advantages over traditional methods such as chair work or desktop-based avatar interactions, as increased presence has been associated with enhanced clinical outcomes.’

In terms of discussing long term benefits of the intervention: As noted by Freeman et al., the effects of VR-based treatments for anxiety disorders, even short-term interventions, have been shown to persist over several years. This highlights the relevance of exploring the potential long-term benefits of VR-based avatar interventions.

As long-term follow-up is not included in the current study, long term effects is discussed in the section on limitations of the study:

‘Although VR-based interventions have demonstrated lasting effects in related fields, such as anxiety disorders ^73^, this study does not include extended follow-up periods. As such, it cannot address the durability of therapeutic gains over time. Future research should incorporate long-term follow-up to evaluate the persistence of this VR-based treatment’s effect.’

3. *Outcome Measures:*

*The selection of outcome measures is comprehensive and encompasses various dimensions of the disorder. However, aligning the outcome measures with internationally recognized standards (e.g., ICHOM: International Consortium for Health Outcomes Measurement) would enable further comparability and applicability of findings to the existing literature (see Austin et al., 2023).*

**Response:**

We very much appreciate the reviewer’s valuable observation regarding alignment with internationally recognized standards, such as those proposed by the International Consortium for Health Outcomes Measurement (ICHOM). We fully agree that the use of standardized outcome measures facilitate comparability across studies. Unfortunately, we were not aware of this set of recommendations by ICHOM and as the study has commenced, we are not able to make changes to the battery at this time.

At the same time, we would like to highlight that the current study is investigating a novel intervention, a VR-based psychotherapy that, to our knowledge, has not previously been applied to eating disorders. Given the exploratory nature of this research and the early stage of evaluating this treatment, we selected a broad and comprehensive set of outcome measures to capture multiple factors that could potentially be impacted by the intervention. These include psychological and behavioral domains relevant to eating disorder psychopathology, as well as potential mechanisms of change specific to avatar therapy (e.g., eating disorder voice experience, change in power balance between the participants and their eating disorder voice). This comprehensive approach allows us to identify potential targets for refinement in future studies and to generate hypotheses for which outcomes are most sensitive to change.

While our outcome battery does not fully align with the ICHOM standard set, it was informed by existing literature on avatar therapy for anorexia nervosa (Thompson et al., 2022), our own feasibility study, clinical expert input, and theoretical considerations related to the intervention’s mechanisms of action.

In the ‘Contributions and Limitations’ section of the Discussion, we added the following to underscore the exploratory nature of many of the outcome measures and the precautions taken in this regard:

‘As the study is experimental, a broad line of explorative outcomes has been included, with the purpose of informing the direction of future studies. These outcomes include participants’ experience of recovery post-intervention, level of emotion regulation, and level of cognitive flexibility. We have also included a range of measures on the power balance between the participants and their eating disorder voice, as well as eating disorder-related symptoms and potential negative effects of the therapy.

The strengths of the study must, however, be seen in the light of the potential limitations. The extensive line of exploratory outcomes may give cause to chance of multiplicity. Therefore, any exploratory findings will be interpreted with caution.’

We noted that the ICHOM recommends the use of the GADS for measuring anxiety symptoms. We chose the HADS depression and anxiety scale, as it is a brief self-report measure (that also works well for outpatients), making it easy to integrate into a comprehensive assessment battery. Additionally, it focuses mostly on the cognitive and emotional aspects of anxiety and depression, excluding somatic symptoms that may overlap with certain depressive symptoms (e.g., fatigue, weight loss, appetite disturbance). This allows for a cleaner measurement of psychological distress and can help reduce confounding by physical symptoms, particularly relevant in eating disorders and depression. However, the use of the HADS does limit comparability with other studies, as it is not widely used in RCT’s with eating disorder populations. We recognize the importance of harmonizing outcomes in the field and plan to reflect on how our findings might be mapped onto standardized outcome frameworks such as those from ICHOM in future dissemination and follow-up research.

4. *Statistical Methods:*

*The use of linear mixed models with repeated measurements is an appropriate choice for analyzing longitudinal data, allowing for robust assessment of intervention effects over time. Your intention-to-treat approach ensures rigor in statistical analysis. However, given the commonly high dropout rates seen in eating disorder interventions, how do the authors anticipate dealing with missing data (e.g., multiple imputation) and any subsequent sensitivity analyses (e.g., to assess the robustness of imputed results). Given the heterogeneity of eating disorder populations across different clinical settings, the authors may want to consider outlining the potential for subgroup analyses.*

**Response**:

Thank you for the positive feedback on our statistical approach and for raising these important considerations regarding the handling of missing data and subgroup analyses.

**Handling of Missing Data and Sensitivity Analyses:**

We have replaced the proposed analysis method of mixed effects models with standard ANCOVA of change from baseline with treatment and baseline value as covariate for the primary and secondary endpoints. Missing data will be multiply imputed under MAR. Departures from this assumption will be examined by employing a tipping point analysis, by progressively worsening the imputed results and examining when the primary analysis conclusion is overturned.

We have clarified these analytical strategies in the statistical methods section, to improve transparency and reproducibility:

‘Analyses will be performed on the ITT population for the primary and secondary endpoints, i.e., analyzing all randomized participants according to the arm they were randomized to. The analysis method will be ANCOVA of change from baseline with treatment as factor and baseline value as covariate at the corresponding post-baseline visit, i.e., month 12. Treatment difference point estimates will be reported together with 95% CIs and p-value. Missing values will be multiply imputed. For the exploratory endpoints, a modified ITT set will be used, requiring at least one post-baseline measurement for the inclusion in the analysis for each endpoint. The analysis method will be linear mixed effects model. Results will be reported for both post-baseline visits. For the primary and secondary endpoints, missing data will be multiply imputed under missing at random (MAR). Sensitivity analyses will involve exploring departures from the MAR assumption, i.e., a tipping point analysis is planned to assess how far must the imputed values deviate from the MAR assumption before the conclusion of the primary analysis is overturned. For the exploratory endpoints, missing data will be implicitly handled by the linear mixed effects model.

**Subgroup Analyses:**

We appreciate the suggestion to consider subgroup analyses, given the heterogeneity often observed in eating disorder populations. In our analysis plan, we will explore potential moderators of treatment effect, including diagnosis subtype (e.g., anorexia nervosa, bulimia nervosa), age group, and treatment setting (inpatient vs. outpatient), if sample sizes permit. Details of model specifications, imputation methods & assumptions, sensitivity analyses and subgroup analyses will be provided in the statistical analysis plan (SAP), that will be made publicly available prior to data lock (i.e., before the dataset is finalized and analysis starts).

**Additional changes made to the manuscript:**

1. **Amendment to the protocol**:

We have made the following update in response to recruitment challenges and to improve transparency in our reporting:

We have outlined this amendment to the protocol (comparator definition) in the section ‘Explanation for the choice of comparators’:

Recruitment has proceeded more slowly than anticipated under our original inclusion criteria. Consequently, we have broadened eligibility and expanded the TAU comparator to include support from private dietitians and consultations with private psychiatrists or general practitioners. This protocol amendment will clearly be documented in the reporting of the trial results.’

In the context of broadening inclusion, we have also added a new means of recruitment, stated in the section ‘Recruitment’:

‘Additionally, we intend to disseminate information regarding the study through social media platforms, and via the online platform trialtree.com, mitigating contact between research projects and potential participants, providing individuals with a diagnosis of eating disorder the opportunity to self-refer. All referrals will be screened for eligibility.….and via the online platform trialtree.com, mitigating contact between research projects and potential participants.’

2. **Supplementary information – AI and user involvement**

In line with new spirit guidelines, as outlined in the *SPIRIT 2025 explanation and elaboration: updated guideline for protocols of randomised trials*, we have added a section on **how we have used AI** in writing the protocol, and a section on how **we have involved patients and plan to involve patients in the development, analysis, and dissemination**.

It has been added to the manuscript that we have used the SPIRIT 2013 guidelines, together with the *SPIRIT 2025 explanation and elaboration: updated guideline for protocols of randomised trials*:

‘The SPIRIT 2013 reporting guidelines has been applied together with the SPIRIT 2025 explanation and elaboration: updated guideline for protocols of randomised trials ^27,28^.’

It now states in a new section named ‘The use of AI’:

**‘The use of AI**

Generative AI (OpenAI’s ChatGPT) was used as a writing support tool during the preparation of the protocol. The tool was used primarily to support reference searching during protocol development and for language editing and restructuring of text to improve clarity. All substantive ideas, research design, hypotheses, and technical and clinical details were developed by the research team and collaborators. All references and scientific claims have been manually verified. No text has been included without review, editing, and approval by the research team. The use of AI has been limited to support drafting and formatting, not content generation or decision-making.’

It now states in a new section on user involvement named ‘Plans for, patient or public involvement in the design, conduct, and reporting of the trial’:

**Plans for, patient or public involvement in the design, conduct, and reporting** **of the trial**

The VR-based avatar intervention of this study was initially co-developed with individuals with a diagnosis of eating disorder (n=8) and clinicians with expertise in eating disorder treatment (n=5), whose input shaped the VR content and therapeutic manual to ensure relevance and acceptability. Additionally, participants in the feasibility study (n=10) delivered feedback on the intervention, further informing the treatment protocol. Feedback on the experience of completing the assessment battery was also provided by an individual with lived experience. The analysis and dissemination phases will also include participants in the trial, ensuring their perspectives in the interpretation and communication of the findings.

3. **Removing mentioning of blood sampling:**

Blood sampling analysis has been removed from the manuscript, including in the ‘outcome-measures section’, as these will be reported separately and not as part of the reporting on the efficacy of the intervention.

4. **Clarification of the therapy setting:**

To ensure that it is clear how the study setting appear, we have added the following in the section on ‘Intervention description’:

‘The therapy is conducted in a shared setting, with both the therapist and participant present in the room, utilizing a computer and VR headset to deliver the intervention.’

5. **Overall changes to the ‘Introduction’ section:**

The **introduction** has been revised in line with reviewer comments, in doing so, changes has been made in a significant portion of the introduction, to avoid repetition:

**‘Introduction**

**Background and rationale**

Eating disorders are characterized by dangerous eating habits and a tendency to overestimate the importance of weight and body shape in one’s self-evaluation ^1^. These illnesses affect at least 7 % ^2^ of the global population and have profound physical and psychological costs for the affected individual ^3^, involving a high risk of early mortality^4,5^.

Additionally, eating disorders are associated with a significant economic burden, including health care costs, reduced employment rates, and loss of productivity. In the European union the annual costs approaches €1 trillion, exceeding the economic impact of anxiety and depression^6^.

First line treatments for eating disorders include psychological therapies^7^, however, many psychological interventions for eating disorders report suboptimal outcomes ^8–10^ and dropout rates have been estimated to range from 20-70% ^11,12^. Even when treatment is completed, relapse rates have been found to be around 30% ^12–14^. Consequently, there is significant interest in developing novel therapies that are engaging and capable of addressing characteristics that may serve as maintaining factors in eating disorder psychopathology.

**The inner voice of the eating disorder, a new target of intervention**

An estimated 94% of patients with an eating disorder report a critical internal voice commenting in second or third person on shape/weight, eating, and self-worth, often referred to as the “eating disorder voice”^15,16^. Patients report that the eating disorder voice emerges at the onset of their illness. It is commonly perceived as supportive initially; however over time, it transforms into a critical and dominant voice, forcing the individual to use destructive eating behaviors ^16^.

Qualitative studies have explained the eating disorder voice’s transformation into a critical and dominating presence as a shift in the relationship between the self and the eating disorder. Initially valued by the person, the disorder becomes a more dissociated and stressful part of the self that overrules the desires and qualities of the ‘true’ self, leaving the individual feeling powerless ^17–19^.

The experience of a more powerful eating disorder voice is associated with increased use of compensatory behaviors (e.g., fasting, vomiting, laxative misuse, compensatory exercise) and longer illness duration ^20^. In line with this, research suggests that the individual’s subordinate position to the eating disorder voice could play a substantial role in maintaining eating disorder symptoms and contribute to challenges in treatment engagement ^20^ Despite its clinical relevance, this power dynamic is rarely addressed directly in standard treatments. Targeting it in therapy may enhance clinical outcomes and treatment engagement by helping patients regain control over the voice and strengthen their identity beyond the disorder ^16,21–23^.

**Avatar-based therapy for eating disorders**

A growing body of research suggests that the experience of an inner eating disorder voice shares a key relational feature with distressing verbal auditory hallucinations, namely the subordinate position that individuals often adopt in relation to the voice ^20–23^. This overlap has prompted interest in applying relational therapies developed for voice-hearing (e.g. voice dialogue), to address the eating disorder voice, an approach supported by emerging qualitative findings ^22,23^.

AVATAR Therapy, a novel relational intervention, originally designed for voice hearing targets this power imbalance. In the therapy, participants engage in real-time dialogue with a digital avatar representing their distressing voice, allowing them to challenge this power dynamic. The intervention has demonstrated efficacy in reducing the severity of the psychotic voice and alleviating associated distress within two fully powered randomized controlled trials^24,25^.Given that AVATAR Therapy directly targets the shared relational dynamic between voice hearers and people with eating disorders, it provides a compelling rationale for testing the intervention’s applicability in eating disorder populations^18,26^.

Based on this, a case-series feasibility study involving 12 participants, tested the application of AVATAR therapy for patients with anorexia nervosa, with the aim of helping participants regain power over their eating disorder voice and reconnect with their identity beyond the eating disorder. ^17^. The treatment approach met the predetermined thresholds for both acceptability and safety ^17^. Furthermore, while not powered as a test of efficacy, the study provided promising indications of potential efficacy in enhancing self-compassion and reducing symptoms of stress and anxiety ^17^. Qualitative data from the study suggested that participants reported increased confidence in asserting themselves against the eating disorder, which contributed to a sense of empowerment to resist its influence. ^17^.

The first AVATAR Therapy for psychosis and anorexia nervosa was delivered on a 2D computer screen. Our research group recently tested the efficacy an immersive 3D avatar intervention for psychosis, mirroring the positive findings from the original AVATAR therapy trial^27^. Building on this, we adapted the virtual reality (VR) software to individuals with eating disorders. In a feasibility study (n=10), this immersive version met the predefined thresholds for feasibility and acceptability and showed promising preliminary treatment effect (unpublished work). Based on these initial findings, the current study aims to investigate the efficacy of an immersive VR-based avatar intervention for eating disorders. The study represents the first fully powered, large-scale, methodologically rigorous clinical trial to explore whether a relational, dialogue-based approach, such as avatar intervention can reduce symptoms and improve quality of life for patients with an eating disorder.

The application of avatar-based intervention to eating disorders is an innovative approach that addresses a critical and underexplored mechanism in eating disorder pathology. By giving form and voice to the internal critical dialogue, avatar-based intervention may facilitate a clearer separation between the individual and the disorder, an important mechanism in the context of eating disorders which are often ego-syntonic and characterized by ambivalence towards change^21,28^. The immersive format of VR-based avatar intervention may support the development of a stronger, recovery-oriented self-narrative; something that traditional talking therapies may struggle to achieve with the same emotional impact. If the study yields positive findings, it could pave the way for new research on psychological interventions for eating disorders. The next step would involve replicating these results across diverse settings.’

**Suggested references from reviewer 3:**

*Schmidt, U., Adan, R., Böhm, I., Campbell, I. C., Dingemans, A., Ehrlich, S., Elzakkers, I., Favaro, A., Giel, K., Harrison, A., Himmerich, H., Hoek, H. W., Herpertz-Dahlmann, B., Kas, M. J., Seitz, J., Smeets, P., Sternheim, L., Tenconi, E., van Elburg, A., van Furth, E., … Zipfel, S. (2016). Eating disorders: the big issue.The lancet. Psychiatry,3(4), 313–315. https://doi.org/10.1016/S2215-0366(16)00081-X*

*Aya, V., Ulusoy, K., & Cardi, V. (2019). A systematic review of the 'eating disorder voice' experience.International review of psychiatry (Abingdon, England),31(4), 347–366. https://doi.org/10.1080/09540261.2019.1593112*

*Natali, L., Ward, T., Rowlands, K., Aya, V., Treasure, J., & Cardi, V. (2023). Changes in the eating disorder voice over time and the association of voice characteristics at baseline with clinical symptoms in patients with anorexia nervosa. Clinical psychology & psychotherapy, 10.1002/cpp.2934. Advance online publication.* [*https://doi.org/10.1002/cpp.2934*](https://doi.org/10.1002/cpp.2934)

*Ward, T., Rus-Calafell, M., Ramadhan, Z., Soumelidou, O., Fornells-Ambrojo, M., Garety, P., & Craig, T. K. J. (2020). AVATAR Therapy for Distressing Voices: A Comprehensive Account of Therapeutic Targets. Schizophrenia bulletin,46(5), 1038–1044. https://doi.org/10.1093/schbul/sbaa061*

*Austin, A., De Silva, U., Ilesanmi, C., Likitabhorn, T., Miller, I., Sousa Fialho, M. D. L., Austin, S. B., Caldwell, B., Chew, C. S. E., Chua, S. N., Dooley-Hash, S., Downs, J., El Khazen Hadati, C., Herpertz-Dahlmann, B., Lampert, J., Latzer, Y., Machado, P. P. P., Maguire, S., Malik, Moser, C.M., Myers, E., Pastor, I.R., Russell, J., Smolar, L., Steiger, H., Tan, E., Trujillo-Chi Vacuán, E., Tseng, M.M., van Furth, E.F., Wildes, J.E., Peat, C., Richmond, T.K.(2023). International consensus on patient-centred outcomes in eating disorders. The lancet. Psychiatry,10(12), 966–973. https://doi.org/10.1016/S2215-0366(23)00265-1*

*Freeman, D., Reeve, S., Robinson, A., Ehlers, A., Clark, D., Spanlang, B., & Slater, M. (2017). Virtual reality in the assessment, understanding, and treatment of mental health disorders. Psychological medicine, 47(14), 2393–2400. https://doi.org/10.1017/S003329171700040X*
